# Draft Genome Assemblies of Two Staphylococcus pseudintermedius Strains Isolated from Canine Skin Biopsy Specimens

**DOI:** 10.1128/MRA.00369-20

**Published:** 2020-05-28

**Authors:** Wenqi Cao, Karly Hicks, Amelia White, Terri Hathcock, Robert Kennis, Dawn Boothe, Dapeng Zhang, Xu Wang

**Affiliations:** a Department of Pathobiology, Auburn University, Auburn, Alabama, USA; b Department of Clinical Sciences, Auburn University, Auburn, Alabama, USA; c Department of Anatomy, Physiology and Pharmacology, Auburn University, Auburn, Alabama, USA; d Department of Biology, Saint Louis University, St. Louis, Missouri, USA; e HudsonAlpha Institute for Biotechnology, Huntsville, Alabama, USA; f Alabama Agricultural Experiment Station, Auburn University, Auburn, Alabama, USA; Indiana University, Bloomington

## Abstract

Staphylococcus pseudintermedius is a Gram-positive bacterial species highly relevant to animal and human health. In this study, we report the draft genome sequences of two clinical isolates of S. pseudintermedius from canine skin biopsy specimens at the Dermatology Service of the Auburn University Small Animal Teaching Hospital.

## ANNOUNCEMENT

Staphylococcus pseudintermedius is a coagulase-positive coccus first identified in 2005 and distinguished from S. intermedius in several growth and biochemical features (1). It is the most commonly isolated pathogen in canine dermatological infections, such as pyoderma, wound infections, and otitis externa (2, 3). Although transmission of S. pseudintermedius between dogs and humans is uncommon, there are several cases indicating risk of zoonotic transmission by direct contact with the cutaneous lesion (4). Since the first case of methicillin-resistant S. pseudintermedius (MRSP) infection emerged in the mid-1980s, the incidence has increased dramatically and become a serious threat to canine health worldwide. MRSP shows resistance to several classes of antimicrobial drugs, and as a result, there are very limited options for clinical therapy (5, 6).

Two strains of S. pseudintermedius, M1S and M3S, were isolated from punch biopsy specimens of lesional skin from two adult dogs with dermatitis, a female American Staffordshire terrier and a female Shih Tzu, respectively. They were acquired in the course of routine clinical patient care and exempt from Institutional Animal Care and Use Committee (IACUC) approval. The isolates were identified as belonging to the Staphylococcus intermedius group using phenotypic tests, including catalase, coagulase, fermentation patterns, and hemolysis, by the Bacteriology and Mycology Laboratory at Auburn University College of Veterinary Medicine. Conventionally, canine S. intermedius group isolates are referred to as S. pseudintermedius (7), and the species identity was confirmed by comparison with a previously published S. pseudintermedius genome (8). The antimicrobial susceptibility profiles were determined using broth microdilution (Vitek 2, bioMérieux, USA) and agar disk diffusion (Table 1). Both isolates displayed multidrug resistance when interpreted using Clinical and Laboratory Standards Institute (CLSI) guidelines (9).

**TABLE 1:** MICs with interpretation for isolates M1S and M3S as determined using CLSI guidelines

Antibiotic	Data for strain:			
	M1S		M3S	
	MIC (μg/ml)	Interpretation	MIC (μg/ml)	Interpretation
Aminoglycosides				
Amikacin	≤2	S^*a*^	4	S
Gentamicin	≥16	R^*b*^	≥16	R
Fluoroquinolones				
Enrofloxacin	≥4	R	≥4	R
Marbofloxacin	≥4	R	≥4	R
Pradofloxacin	1	I^*c*^	1	I
Tetracyclines				
Doxycycline	≥16	R	8	R
Minocycline	8	R	2	R
Macrolide				
Erythromycin	≥8	R	≥8	R
Lincosamide				
Clindamycin	≥4	R	≥4	R
Phenicol				
Chloramphenicol	8	S	8	S
Rifamycin				
Rifampin^*d*^		S		S
Beta-lactam				
Oxacillin^*d*^		R		R
Sulfonamide				
Trimethoprim-sulfamethoxazole	≥320	R	≥320	R

a S, susceptible.

b R, resistant.

c I, intermediate.

d Tested by agar disk diffusion.

Prior to sequencing, the isolates were recovered by plating onto 5% bovine blood agar and incubating at 37°C in 5% CO_2_ for 18 to 24 h. The isolates were twice subcultured to ensure viability and purity. DNA was extracted using an AllPrep PowerFecal DNA/RNA kit (Qiagen, MD) and quantified by Qubit fluorometer (Invitrogen). One microgram of genomic DNA was fragmented by an M220 focused ultrasonicator with a 500-bp targeted insert size (Covaris, MA). DNA libraries were constructed using a NEBNext Ultra II DNA library prep kit for Illumina (New England Biolabs, MA). The libraries were sequenced on an Illumina NovaSeq 6000 machine.

In total, 27,210,622 and 30,500,546 150-bp paired-end reads were generated for M1S and M3S, respectively, and the quality was assessed by FastQC (10). Adapters and low-quality bases were removed using Trimmomatic v0.39 (11). *De novo* assembly of the bacterial genome was performed using MEGAHIT 1.2.9 (12), resulting in a 2,818,651-bp M1S assembly (37 contigs; *N*_50_, 176,632 bp; G+C content, 37.1%) and a 2,736,991-bp M3S assembly (43 contigs; *N*_50_, 136,323 bp; G+C content, 37.3%). The CheckM v1.1.2 (13) completeness is 99.43% for both genomes. Genome coverage was estimated to be 1,380.8× (M1S) and 1596.8× (M3S) using Seqkit v1.2-r94 (14). Shorter and lowly covered contigs (<5 kb; depth, <15×) were removed. Plasflow v1.1.0 was used to predict plasmid sequences in these genomes (15). Genome annotation was performed using the Prokka pipeline v1.14.6 (16) and the NCBI Prokaryotic Genome Annotation Pipeline (17). In all, 2,760 gene models were predicted in the M1S genome and 2,660 in M3S. Default parameters were used except where otherwise noted.

### Data availability.

The assemblies were deposited at DDBJ/ENA/GenBank under the accession numbers JAAXMN000000000 and JAAXMO000000000. Raw sequencing data are available in the NCBI Sequence Read Archive under the accession numbers PRJNA623239 and PRJNA623240.
